# Prenatal depressive symptoms in Latinas: a qualitative investigation

**DOI:** 10.3389/fgwh.2024.1458157

**Published:** 2024-11-18

**Authors:** Isabel F. Almeida

**Affiliations:** Department of Chicano/Latino Studies, University of California, Irvine, CA, United States

**Keywords:** Latinas, prenatal depression, pregnancy, maternal mental health, maternal depression

## Abstract

**Introduction:**

Exposure to prenatal depressive symptoms is associated with an increased risk of adverse pregnancy outcomes and child health complications. Research examining experiences of maternal depression among Latinas living in the United States, who have increased risk for experiencing prenatal depression symptoms, is lacking.

**Objectives:**

The purpose of this qualitative investigation is to examine the experience of prenatal depression symptoms among Latinas primarily of Mexican descent.

**Methods:**

Fourteen pregnant Latinas shared their experiences of depressive symptoms during pregnancy in individual interviews and one focus group conducted in Spanish.

**Results:**

The most described symptoms of depression were periods of crying for no reason; feelings of irritability, sadness, and loneliness; and a loss of interest in normal activities. The participants coped with their depressive symptoms through distraction. Additionally, participants shared their beliefs that experiencing prenatal depressive symptoms was normal due to hormonal changes and that social support was protective. Themes about the baby “feeling” the mother's emotions during pregnancy and that prenatal depression is misunderstood also emerged.

**Conclusions:**

These findings shed light on how Latina's experience maternal depressive symptoms and call for additional research on risk factors during the perinatal period among this growing subpopulation.

## Background

Between 8.5% and 11% of women living in the United States (US) experience depressive symptoms during pregnancy (1). Exposure to prenatal depressive symptoms has been associated with adverse pregnancy and child outcomes such as low birth weight, premature birth, slow fetal growth rates, maternal anemia and diabetes, and hypertensive disorders (2, 3). The consequences of prenatal depression persist from infancy to early development. For example, infants born to mothers who experienced depression during pregnancy have a 13% greater incidence of preterm birth and a 15% greater incidence of low birth weight (4). Moreover, a systematic review revealed that children with mothers with prenatal depression were six times more likely to experience delayed emotional development and five times more likely to undergo delayed language development (5).

Racial and ethnic disparities in maternal health outcomes constitute a major public health challenge in the US. Women of color have a higher risk of experiencing depression during pregnancy than their nonminority counterparts (6, 7). Latinos comprise 19.5% of the total U.S. population (8), and their birth rate is 31% higher than the national average (9, 10). However, most studies on maternal mental health have focused on non-Hispanic White person(s)/people, middle-class, and well-educated samples. Research shows that Latinas living in the US are uniquely vulnerable to sources of distress, including immigration-related stressors, discrimination, evolving cultural orientations, inaccessibility to health services, and financial pressure that, when experienced during pregnancy, can alter their birth outcomes (11–13).

Most research on maternal mental health among Latinas has focused on identifying the cultural (e.g., generational status, acculturative stress, border-crossing trauma, and discrimination) and contextual (e.g., poverty, childhood abuse, and domestic violence) risk factors of *postpartum* depression (11, 14). However, less is known about depressive symptoms among Latinas *during* pregnancy. More recently, stressors such as discrimination have been recognized as contributors to depressive symptoms among Latinas (15). Harris and colleagues (15) indicate that during pregnancy, experiences of discrimination may result in worry and self-blame, which often precede depressive symptoms. Stigma and inferior treatment associated with poverty are also linked with prenatal depressive symptoms, which could be particularly salient for immigrant Latina mothers who may feel powerless to advocate for themselves due to fears of job loss or deportation (15).

In another study involving a sample of pregnant Mexican-American women, acculturative stress and perceived stress—but not acculturation or discrimination—were found to be linked with elevated depressive symptoms (16). Data from this study suggest that conflicts in cultural values, including those concerning traditional gender roles and material success, may not align in acculturated individuals, thus contributing to conflict and distress during this transitional period.

Financial hardships, specifically household food insecurity, have also been associated with elevated levels of depressive symptoms during the second and third trimesters of pregnancy among Latinas (17). Authors theorize that food insecurity may boost the level of maternal stress during pregnancy, contributing to hypothalamic-pituitary-adrenocortical axis dysfunction that can increase the risk for depression. In another study involving a large sample of 480 predominantly low-income Latinas, adverse childhood experiences (ACEs) have been similarly linked with an increased likelihood of probable depression during pregnancy (18). This pattern was evident among Latina mothers who had experienced four or more ACEs.

More generally, values, beliefs, and social norms shape the cultural relevance of mental health within Latino communities (19, 20). Barriers such as stigma around mental illness, distrust of the medical system, limited access to health insurance and services, and linguistic issues (e.g., miscommunication) can impede access to mental health services (21, 22). Research that explores these cultural and contextual factors is critical to address the mental health needs of the growing Latino population.

### The present study

Several questions regarding the experience of prenatal depression among Latinas living in the US remain unanswered. Thus, the purpose of this qualitative investigation is to thoroughly examine the experience of prenatal depression among Latinas, given the research gap and the strong associations of prenatal depression with adverse outcomes. In particular, the aim of this study is to qualitatively investigate the (1) experience of prenatal depression among Latinas, (2) the perceptions and beliefs about prenatal depression, and (3) the ways in which the Latinas' families have responded to these women's depressive experiences.

## Methods

### Participants

This qualitative study includes 11 individual face-to-face interviews and one focus group discussion involving three participants. To be eligible for the study, participants had to be pregnant and 18 years of age or older, self-identify as Latina or Hispanic, and be fluent in Spanish. The participants were financially compensated for their time with $40 gift cards.

### Recruitment strategy

The participants were recruited through flyers, word of mouth, and personal communication in the waiting room of a prenatal clinic in West Los Angeles, California. Half of the participants in the study were recruited at the prenatal clinic, and the other half were recruited using flyers, referrals, and word of mouth.

### Interview guide development

The interview guide was translated from English to Spanish, and it included questions about the participants' personal experiences and feelings related to depression during their current pregnancy and their general beliefs and attitudes about prenatal depression. The participants were also asked to comment on their families' beliefs about prenatal depression. This guide was informed by cultural psychology and social science research. Given the sensitivity of the questions, the researchers ensured the protection of the participants' well-being. For example, the participants were offered referral contact information for mental health hotlines and counseling services in the area, including services with multilingual staff and those with free and low sliding-scale fee options. Additionally, a clinical psychologist with expertise in Latino mental health was available for consultation in the case of an adverse event during the interview.

### Data collection and transcription

Focus group discussions and individual interviews were conducted in Spanish. The focus group discussions were held at a prenatal medical clinic in West Los Angeles, and the individual interviews were conducted either at the prenatal medical clinic or at local coffee shops. Individual interviews lasted 33 min on average and ranged from 21 to 49 min, whereas the focus group discussions lasted for one hour. More individual interviews and fewer focus group discussions were conducted than originally anticipated due to scheduling difficulties. The research team sought to increase the dependability of the data by interviewing participants until data saturation was reached after the 11th interview and by taking field notes of all individual interviews and focus group discussions. The focus group discussions and the individual interviews were audio-recorded and transcribed by the author of the manuscript. All the transcripts were reviewed for accuracy by a research assistant.

### Data analysis

Data were analyzed following the six-phase thematic analysis developed by Braun and Clarke (23). Thematic analysis entails the coding of all the data into themes and patterns. In the first phase, the author and a research assistant read the entire data set twice, noting initial ideas, similarities, and differences in the interviews. Face-to-face meetings were held to discuss their notes.

In the second phase, researchers generated initial codes by tagging selected pieces of text using the qualitative software ATLAS.ti [Version 8 for Mac; (24)]. The researchers independently coded the features of two randomly selected interview transcripts. Initial codes were established through deductive reasoning, which involves approaching the data with preconceived ideas and potential codes based on existing knowledge, which are then explored within the data set (25). The transcripts were uploaded in Spanish and the codes were written in English.

The codes from the transcript review were consolidated into an initial codebook. The team refined the codebook during meetings, and discrepancies were resolved through discussions. To maintain data quality through triangulation, the two researchers independently coded the interview transcripts, comparing their interpretations to identify consistencies and discrepancies. The coders reached an interrater reliability of 91% (26).

In the third phase, the research team grouped relevant data for potential themes. The codes were analyzed and organized into code groups, initial overarching themes, and subthemes. Consistent with the key principles of data triangulation, three outside qualitative researchers were consulted to help interpret the data from different perspectives. The research team subsequently refined the themes and subthemes during the fourth phase. This phase involved checking whether the themes worked in relation to the codes and the entire data set, splitting and collapsing the themes as seen fit (23). In the fifth phase, the themes were finalized, and direct quotes were selected to provide evidence during the sixth phase of the thematic analysis. For the purposes of this manuscript, the quotations were translated into English by a bilingual member of the study team.

### Ethical considerations

The researchers obtained verbal consent for participation in the study. The participants were provided with study information sheets containing the research purpose, procedures, potential risks and benefits, compensation, confidentiality measures, right to withdraw from the study, and the principal investigator's contact information. Data were kept confidential on password-protected devices and were not shared with anyone who was not directly involved in the study. The participants' identities were anonymized through pseudonyms, ensuring that no link emerged between data and individuals. There were no potential conflicts of interest to be disclosed. The Institutional Review Board of the University of California, Los Angeles, approved all study materials and procedures (IRB#:18-001850).

### Measures

The participants were asked to complete a demographics questionnaire upon finishing the individual interviews or focus group discussions. They were instructed to indicate their age, educational attainment, household income, country of birth, parental country of birth (if the participant was born in the US), length of time of living in the US (if the participant was born outside the country), marital status, number of children, and gestational length at the time of data collection.

## Results

### Sample description

The participants' ages ranged from 20 to 39 (*M* = 28.71, *SD* = 5.35), and the median and mode were 28 years of age. Half of the participants had completed high school, and the other half had college degrees. The average annual household income was $41,157 (*SD* = 47,650), with a range from $1,200 to $180,000, a median of $23,000, and a mode of $20,000.

Slightly more than half of the participants in the sample were foreign-born (*n* = 8). Among the participants who were born outside the US, six participants were born in Mexico, one in Guatemala, and one in Honduras. They had been living in the US for 17 years on average, with a range from seven to 35 years, a median of 16.5 years, and a mode of 15 years. All of the parents of the U.S.-born participants (*n* = 6) were of Mexican origin.

Most of the participants in the sample were either married (*n* = 8) or not married but in a romantic relationship (*n* = 5), with the remaining participant neither married nor engaged in a romantic relationship. Majority of the participants had previously given birth (*n* = 10). The participants' number of previous children ranged from zero to four, with a median and mode of one child. At the time of their interview or participation in the focus group, the participants' average length of gestation was 25.39 weeks (*SD* = 9.18), with a range from 7.5 weeks to 38 weeks, a median of 25.5 weeks, and modes of 25 and 33 weeks. The participants' demographic information is presented in Table 1.

**Table 1:** Sample demographic information (*N* = 14).

Pseudonym	Age	Education	Income	Place of birth	Years living in the U.S.	Relationship status	Number of children	Length of gestation (weeks)	Interview format
Delia	39	HS	20,000	Mexico	15	1	3	25	Focus group
Flor	35	HS	20,000	Guatemala	15	2	4	26	Focus group
Andrea	28	Associate's	23,000	U.S.	N/A	2	1	14	Focus group
Daimí	38	HS	50,000	Honduras	24	1	3	35	Individual Interview
Estela	27	HS	1,200	Mexico	16	2	1	31	Individual Interview
Amelia	28	HS	2,000	Mexico	17	1	3	38	Individual Interview
Martha	28	HS	23,000	Mexico	7	1	2	33	Individual Interview
Victoria	28	Bachelor's	100,000	Mexico	25	1	0	25	Individual Interview
Melida	29	Bachelor's	180,000	U.S.	N/A	1	0	23	Individual Interview
Anna	23	HS	40,000	U.S.	N/A	3	0	12	Individual Interview
Carmen	25	Bachelor's	60,000	U.S.	N/A	2	0	33	Individual Interview
Carla	20	Associate's	30,000	U.S.	N/A	1	1	7.5	Individual Interview
Natasha	25	Associate's	12,000	Mexico	20	2	1	21	Individual Interview
Ruby	29	Bachelor's	15,000	U.S.	N/A	1	1	32	Individual Interview

HS, high school. Income is average annual household income. All of the parents of the U.S.-born participants (*n* = 6) were born in Mexico. Relationship status: married = 1; not married but in a romantic relationship = 2; not married and not in a romantic relationship = 3. Length of gestation was measured at the time of their interview or participation in the focus group.

### Themes

Two overarching themes (i.e., depressive symptoms during pregnancy and perceptions and beliefs about depression during pregnancy) and six subthemes (symptomatology, coping through distraction, depressive symptoms during pregnancy are normal, prenatal depression is misunderstood, social support is protective against prenatal depression, and the baby feels the mother's emotions in the womb) emerged from the data (see Table 2).

**Table 2:** Qualitative themes and sub-themes.

Theme	Sub-theme
1. Depressive symptoms during pregnancy	Symptomology
	Coping through distraction
2. Perceptions and beliefs about depression during pregnancy	Depressive symptoms during pregnancy are normal
	Prenatal depression is misunderstood
	Social support is protective against prenatal depression
	Baby feels the mother's emotions

### Theme 1: depressive symptoms during pregnancy

#### Subtheme 1: symptomology

One participant reported being depressed in a previous pregnancy, and most participants (*n* = 9) reported experiencing depressive symptoms at the time of the data collection or at some point during pregnancy. The most commonly described symptoms were bouts of crying for no reason; feelings of irritability, sadness, and loneliness; and loss of interest in normal activities such as leaving their house or seeing their friends. For example, Amelia described pregnancy as “emotionally terrible. I cry for two or three days straight, for no reason, and I am always irritated for no reason. It's miserable. I am not happy, especially in the past few weeks.”

Some participants also noted that their emotions were “all over the place.” For instance, Delia indicated that “the beginning of my pregnancy was different emotionally. I was a bit depressed, and I was crying. I had a lot of emotions. I [seemed to] worry a lot.” These symptoms lasted anywhere from a few hours to a month*.* Anna described her symptoms and experience as follows:

Some days I want to vent and cry for no reason. Sometimes, I am home alone, and I feel very sad and start to cry. I stay in my room, and I don't want to come out. My friends call me to go out with them, but I don't want to, and I block them. [Such emotion] lasts for hours, days, or weeks. This time, I blocked everyone for a month. I didn't want to talk to anyone.

#### Subtheme 2: coping through distraction

Most participants coped with their depressive symptoms through distraction by taking walks, talking to neighbors, watching television, and thinking about something else until the feeling had passed. For example, Daimi shared, “There are times when I feel sad, but I think about something else, and [the feeling] passes. It's something that I can control.” Martha illustrated a similar experience:

Sometimes, I feel sad, and I am really scared to have another baby, but I try to go for a walk, and [the sadness] goes away. If I stay in the house, the stress comes, but I try to go for a walk or talk to my neighbor and [the stress] goes away.

### Theme 2: perceptions and beliefs about depression during pregnancy

#### Subtheme 1: depressive symptoms during pregnancy are normal

According to the participants, they believed that feeling sad or depressed during pregnancy was normal because of the hormonal changes that their bodies were undergoing. As Delia stated, “I think it's normal because there is an evolution of hormones inside you.” Andrea expressed a similar viewpoint, indicating that “[feeling sad or depressed] is normal because your body is changing; my hormones drive me crazy.” Melida concurred, adding that feeling sad or depressed during pregnancy—especially thereafter—is a normal one. According to some participants, their doctors explained that their fluctuating hormone levels may affect their moods throughout pregnancy. Ruby shared the following perspective:

I think that [the feeling of sadness or depression] is normal because the doctors explain to you that your hormones are changing a lot. Suddenly you feel angry and sad, and then you feel happy, or you are crying. The doctor tells you that your emotions will change a lot during all of pregnancy. [Being] happy is a normal [feeling]. If you are sad, then it is also a normal [feeling] because your hormones are completely changing.

Delia likewise acknowledged that her depression and anger were fueled by the hormonal changes that her body was undergoing during pregnancy. As she stated,

When I would get depressed or become angry, I understood that [the cause was] my hormones. I would ask my baby for forgiveness [because] it's not my baby's fault; all the things that are going on in my body [are to blame]. There were days that I didn't even enjoy my favorite food or be with my family. I didn't want anything that used to make me happy. I understood that I was depressed, and I knew that my hormones [were the underlying reason].

#### Subtheme 2: prenatal depression is misunderstood

Many participants stated that their families, partners, and friends did not understand their depressive symptoms and claimed that their feelings were taken lightly. For example, Anna shared, “Instead of understanding me, my boyfriend tells me not to have these feelings [of sadness] because he doesn't want anything to happen to the baby.” When speaking about her family, Karla contended that “they don't really give [my feelings] much attention. They say [that such feelings] will go away. They act as if [what I am experiencing] is nothing.” Furthermore, the participants indicated that they sometimes felt judged and criticized. As Delia stated, “most of my friends understand, but some tell me that [the feeling] is all in my head [and dissuade me from] thinking like that. It's not easy to deal with [this emotion]. You feel guilty when they tell you that.” Amelia shared a similar experience:

I am not really open with my family because they criticize and judge too much. They don't understand, [and] they want everything to be perfect. I have felt judged by my husband. He says that I shouldn't feel depressed, but men don't understand what we as women feel. People need to understand that there will be hard times. Many people don't understand, and they judge you because perhaps they have never been depressed.

#### Subtheme 3: social support is protective against prenatal depression

According to some participants, social support helped protect them from depression during pregnancy. For example, Natasha revealed that “I haven't felt sad or depressed yet. I have enough support. Several people get depressed or anxious because they don't have enough support, but not me.” For Delia, the social support she obtained from her family and church congregation was particularly beneficial:

Seeing how much support I received during pregnancy, everything that everyone did for me to feel good—my husband, my family, my husband's family, and my congregation—it has been incredible. [Such support] helped surmount everything that I was feeling. They called me, were happy for me, and made me see everything positively. That's how I overcame all the bad feelings that I had.

#### Subtheme 4: the unborn baby feels the mother's emotions

Another theme that emerged from the data was the view that the unborn baby could sense the mother's emotions. Many participants stated that they should feel happy to ensure that they were not putting their unborn babies at risk because they believed that “the baby feels everything.” Delia indicated that “I have felt depressed, sad, frustrated, and I've cried. At first, I felt very mad, and I would worry because I didn't want to put my baby at risk.” Amelia believed that if she frequently cried during pregnancy, then her unborn baby would cry as well, noting that her unborn baby experienced all of the emotions that she was feeling during pregnancy. Natasha similarly shared, “All the feelings we have, we are passing on to our [unborn] baby; whatever you feel, the baby feels [as well].”

## Discussion

### Summary of results

This study explored the perceptions of and experiences with prenatal depression among Latinas living in the US who were primarily of Mexican descent and were living in a large urban city. Most participants reported experiencing depressive symptoms during pregnancy. The most frequently reported symptoms included crying without a clear reason and heighted levels of irritability, sadness, and loneliness. The participants also noted a general loss of interest in daily activities. They coped with these symptoms by intentionally shifting their attention away from these emotions via through activities such as outdoor walks and a redirection of their thoughts. Some participants also expressed that support from family and friends helped protect against depressive symptoms.

Most participants also believed that experiencing negative emotions during pregnancy was normal, attributing mood changes to hormonal fluctuations. They also reported feeling misunderstood, judged, or criticized for experiencing these symptoms of depression, which led to feelings of guilt.

### Depressive symptoms during pregnancy

Untreated depression during pregnancy increases the risk of adverse outcomes, including low birth weight and premature birth (2, 3). Latinas often underutilize mental health services and encounter linguistic and structural barriers to accessing mental health treatment (27), thereby increasing the risk of untreated depression. Our findings highlight the need to understand how depressive symptoms manifest during the transition to motherhood of this subgroup of women. Consistent with past research, we found that excessive crying is among the most commonly reported somatic symptoms (15, 28).

This finding aligns with research indicating that Latinas are more likely to present somatic symptoms than mood symptoms because they are more culturally acceptable (29), which can complicate the diagnosis (30). In pregnancy, specifically, one review found that Latinas with depression are more likely than White person(s)/people to report somatic symptoms (31). Further research is needed to understand how these symptoms evolve throughout pregnancy and identify the related risk factors in this subgroup of women.

Future studies should also consider the impact of ACEs on depressive symptomatology within this subgroup. Prior research shows a link between higher ACE scores and the risk of developing maternal depression and the duration of symptoms (32, 33). Especially among Latinas, the experience of four or more ACEs and subdomains of abuse, neglect, and household dysfunction has been associated with the increased risk of maternal depression (18). The qualitative exploration of these experiences may provide insights into the subjective ways that traumatic events in childhood may shape mental health experiences during major life transitions such as pregnancy.

### Perceptions and beliefs about depression during pregnancy

The participants viewed depressive symptoms during pregnancy as normal, attributing them to typical hormonal changes. To our knowledge, this study is the first one to report such findings, although other qualitative studies have indicated that pregnant Latinas recognize maternal depression as a significant issue in their communities (28). The participants also believed that maternal depression is misunderstood and that their feelings were taken lightly, which aligns with findings that depression is often stigmatized within Latino communities (34). This stigma is particularly relevant, as it has been linked with lower engagement in and quality of mental health care among Latinos (35).

The participants reported feeling misunderstood and judged but also acknowledged that social support protected them from negative emotional experiences. These results are consistent with studies highlighting the protective role of social support for mental health during the perinatal period both generally (36, 37) and specifically among Latinas (38, 39). Nonetheless, the role of perceived social support and the extent to which it provides a buffer against prenatal depressive symptoms require further investigation. Future investigations should examine the specific functions and circumstances in which various sources of support—including that of the partner, family, greater social network, and intergenerational influences—mitigate the effects of prenatal depression and promote healthy pregnancy among this subgroup of women.

The participants in this study believed that their emotions could be felt by their unborn babies, a view that aligns with the findings from Sandman et al. (40), which suggest that a mother's psychological state—particularly depressive experiences—may be communicated to the fetus. In response, pregnant Latinas reported actively trying to “feel happy” to avoid potential risks to their unborn babies. These emotion regulation efforts may reduce maternal distress during this sensitive period in their lives, benefiting the health of both the mother and the unborn child (41). Future research could build on these findings to explore the potential pathways in which perinatal emotion regulation strategies impact health outcomes for mothers and children.

### Limitations

This study is not without limitations. Our study is based on a small sample of pregnant Latinas living in a large urban area in Southern California. Additionally, our sample featured an overrepresentation of participants of Mexican heritage, and no specific criteria were applied to diversify the studied population. Thus, our findings cannot be generalized to Latina mothers who reside in the rural areas of the US or originate from Caribbean and Central and South American countries, who may have different cultural beliefs about and experiences with mental health. Latina mothers living in the US are a heterogenous group, with varied lived experiences shaped by factors such as immigration history, cultural traditions, and socioeconomic and generational status (42). Future studies should use comprehensive research strategies to include a wider range of backgrounds within the Latina population to better capture these diverse perspectives and variations in the identified themes.

In addition, our study design does not capture how depressive symptoms may change throughout pregnancy and into postpartum nor identifies the factors that might predict these fluctuations. Such data could offer a fuller understanding of symptom severity and determine the specific periods when depressive symptoms may intensify or diminish. This information could help identify the optimal timing for intervention efforts. Relatedly, follow-up data regarding the sample's obstetric, delivery, or neonatal complications were not collected; this information is important to further understand the context in which these emotional experiences occur.

Moreover, this study relied on self-reported symptoms rather than clinical assessments to identify the symptoms of depression, which could impact the interpretation and applicability of our findings. Thus, future studies should consider the use of clinical diagnostic tools to provide a more accurate understanding of depressive symptoms, allowing for a clearer identification of at-risk groups.

Finally, our data could have been influenced by the decision to conduct fewer focus group discussions and more individual interviews than anticipated. Nonetheless, the research team made every effort to accommodate the needs of the participants, who stated that unreliable transportation, childcare challenges, and work schedules impeded their participation in a focus group. Therefore, individual interviews allowed for more flexibility because they were scheduled at a place and time that were convenient for the participants.

## Conclusion

The findings from this study provide a new understanding of the perceptions of and experiences with prenatal depression among Latinas. Our results highlight several themes that may be essential to the evaluation of the perinatal emotional experiences of Latinas, such as the most prevalent somatic symptoms, the stigma of prenatal depression, and emotion regulation strategies for protecting the expectant mother and her child.

The general state of the literature on maternal mental health has made considerable progress, yet gaps remain in research involving women from historically underrepresented groups in perinatal mental health studies. Very few studies have examined the experiences of Latinas who are at risk for prenatal depression compared to their nonminority counterparts. Efforts to improve their prenatal mental health outcomes are critical; if these results are replicated in larger samples, then they may support the development of targeted interventions to reduce prenatal depression and improve the pregnancy experience of the growing Latina population.

## Data Availability

The raw data supporting the conclusions of this article will be made available by the authors, without undue reservation.
